# Development and validation of a classification approach for extracting severity automatically from electronic health records

**DOI:** 10.1186/s13326-015-0010-8

**Published:** 2015-04-06

**Authors:** Mary Regina Boland, Nicholas P Tatonetti, George Hripcsak

**Affiliations:** Department of Biomedical Informatics, Columbia University, New York, NY USA; Observational Health Data Sciences and Informatics (OHDSI), Columbia University, 622 West 168th Street, PH-20, New York, NY USA; Department of Systems Biology, Columbia University, New York, NY USA; Department of Medicine, Columbia University, New York, NY USA

**Keywords:** Electronic Health Records, Phenotype, Health status indicators, Data mining, Outcome assessment (Health Care)

## Abstract

**Background:**

Electronic Health Records (EHRs) contain a wealth of information useful for studying clinical phenotype-genotype relationships. Severity is important for distinguishing among phenotypes; however other severity indices classify patient-level severity (e.g., mild vs. acute dermatitis) rather than phenotype-level severity (e.g., acne vs. myocardial infarction). Phenotype-level severity is independent of the individual patient’s state and is relative to other phenotypes. Further, phenotype-level severity does not change based on the individual patient. For example, acne is mild at the phenotype-level and relative to other phenotypes. Therefore, a given patient may have a severe form of acne (this is the patient-level severity), but this does not effect its overall designation as a mild phenotype at the phenotype-level.

**Methods:**

We present a method for classifying severity at the phenotype-level that uses the Systemized Nomenclature of Medicine – Clinical Terms. Our method is called the *C*lassification *A*pproach for *E*xtracting *S*everity *A*utomatically from Electronic Health *R*ecords (*CAESAR*). CAESAR combines multiple severity measures – number of comorbidities, medications, procedures, cost, treatment time, and a proportional index term. CAESAR employs a random forest algorithm and these severity measures to discriminate between severe and mild phenotypes.

**Results:**

Using a random forest algorithm and these severity measures as input, CAESAR differentiates between severe and mild phenotypes (sensitivity = 91.67, specificity = 77.78) when compared to a manually evaluated reference standard (*k = 0.716*).

**Conclusions:**

CAESAR enables researchers to measure phenotype severity from EHRs to identify phenotypes that are important for comparative effectiveness research.

## Background

Recently, the Institute of Medicine has stressed the importance of Comparative Effectiveness Research (CER) in informing physician decision-making [1]. As a result, many national and international organizations were formed to study clinically meaningful Health Outcomes of Interest (HOIs). This included the Observational Medical Outcomes Partnership (OMOP), which standardized HOI identification and extraction from electronic data sources for fewer than 50 phenotypes [2]. The Electronic Medical Records and Genomics Network (eMERGE) [3] also classified some 20 phenotypes, which were used to perform Phenome-Wide Association Studies (PheWAS) [4]. However, a short list of phenotypes of interest remains lacking in part because of complexity in defining the term phenotype for use in Electronic Health Records (EHRs) and genetics [5].

EHRs contain a wealth of information for studying phenotypes including longitudinal health information from millions of patients. Extracting phenotypes from EHRs involves many EHR-specific complexities including data sparseness, low data quality [6], bias [7], and healthcare process effects [8].

Many machine-learning techniques that correlate EHR phenotypes with genotypes encounter large false positive rates [3]. Multiple hypothesis correction methods aim to reduce the false positive rate. However, these methods strongly penalize for a large phenotype selection space. A method is needed that efficiently reduces the phenotype selection space to only include important phenotypes. This would reduce the number of false positives in our results and allow us to prioritize phenotypes for CER and rank them by severity.

To extract phenotypes from EHRs, a specialized ontology or terminology is needed that describes phenotypes, their subtypes and the various relationships between phenotypes. Several ontologies/terminologies have been developed for studying human phenotypes including the Human Phenotype Ontology (HPO) [9]. The HPO contains phenotypes with at least some hereditary component, e.g., Gaucher disease. However, EHRs contain phenotypes that are recorded during the clinical encounter that are not necessarily hereditary. To capture a patient’s phenotype from EHRs, we will utilize an ontology specifically designed for phenotype representation in EHRs called the Systemized Nomenclature of Medicine – Clinical Terms (SNOMED-CT) [10,11]. SNOMED-CT captures phenotypes from EHRs, including injuries that are not included in the HPO. Furthermore, SNOMED-CT can be used to capture more clinical content then *International Classification of Diseases, version 9* (ICD-9) codes [12], making SNOMED-CT ideal for phenotype classification. Using SNOMED-CT enables development of a standardized approach that conforms to OMOP’s guidelines promoting data reuse.

Robust methods are needed that address these challenges and reuse existing standards to support data sharing across institutions. This would propel our understanding of phenotypes and allow for robust CER to improve clinical care. This would also help pave the way for truly translational discoveries and allow genotype-phenotype associations to be explored for clinically important phenotypes of interest [13].

An important component when studying phenotypes is phenotype severity. Green et al. demonstrate that a patient’s disease severity at hospital admission was crucial [14] when analyzing phenotype severity at the patient-level. We are interested in classifying phenotypes as either severe or mild at the phenotype-level, which differs from the vast literature on patient-specific severity. Classifying severity at the phenotype-level involves distinguishing acne as a mild condition from myocardial infarction as a severe condition. Contrastingly, patient-level severity assesses whether a given patient has a mild or severe form of a phenotype (e.g., acne). Importantly, phenotype-level severity is independent of the individual patient’s state and is relative to other phenotypes (e.g., acne vs. myocardial infarction). Further, phenotype-level severity does not change based on the individual patient. For example, acne is mild at the phenotype-level, which is relative to other phenotypes. Therefore, a given patient may have a severe form of acne (i.e., patient-level severity = severe), but the overall phenotype-level severity is mild because phenotype-level severity is relative to other phenotypes and does not change based on an individual patient’s patient-level severity.

Studying phenotype severity is complex. The plethora of medical conditions is mirrored by an equally diverse set of severity indices that run the full range of medical condition complexity. For example, there is a severity index specifically designed for nail psoriasis [15], insomnia [16], addiction [17], and even fecal incontinence [18]. However, each of these indices focuses on classifying patients as being either a severe or mild case of a given condition (e.g., psoriasis). They do not capture the difference at the phenotype-level.

Other researchers developed methods to study patient-specific phenotype severity at the organismal level. For example, the Severity of Illness Index assesses patient health using seven separate dimensions [19] consisting of: 1) the stage of the principal diagnosis at time of admission; 2) complications; 3) interactions (i.e., the number of patient comorbidities that are unrelated to the principal diagnosis); 4) dependency (i.e., the amount of care required that is above the ordinary); 5) non-operating room procedures (i.e., the type and number of procedures performed); 6) rate of response to therapy; and 7) remission of acute symptoms directly related to admission.

The Severity of Illness Index is useful for characterizing patients as severe or mild types of a given disease phenotype. However, it does not measure severity at the phenotype-level (e.g., acne vs. myocardial infarction), which is required to reduce the phenotype selection space to only the most severe phenotypes for CER.

In this paper, we describe the development and validation of a *C*lassification *A*pproach for *E*xtracting *S*everity *A*utomatically from Electronic Health *R*ecords (*CAESAR*). CAESAR incorporates the spirit of the Severity of Illness Index, but measures phenotype-level severity rather than patient-level severity. CAESAR was designed specifically for use with EHR-derived phenotypes.

## Methods

### Measuring severity

We used five EHR-specific measures of condition severity that are related to the 7 dimensions from Horn’s patient-level severity index [19] because EHRs differ from research databases [20]. Columbia University Medical Center’s (CUMC) Institutional Review Board approved this study.

*Condition treatment time* can be indicative of severity and so it was included as a severity measure. Treatment time is particularly indicative of severity for acute conditions, e.g., fractures, wounds or burns, because minor (less severe) fractures often heal more rapidly than major fractures (more severe). However, treatment time is also dependent on the chronicity of the disease [21], which is separate from severity. Treatment time can also have other effects when recorded in EHRs [22-24].

Because hospital duration time can be influenced by many factors, e.g., patients’ other comorbidities, we decided to analyze the condition treatment time. While inter-dependent, hospital duration time is typically a subset of the entire condition treatment time (which can include multiple hospital visits).

**Figure 1:**
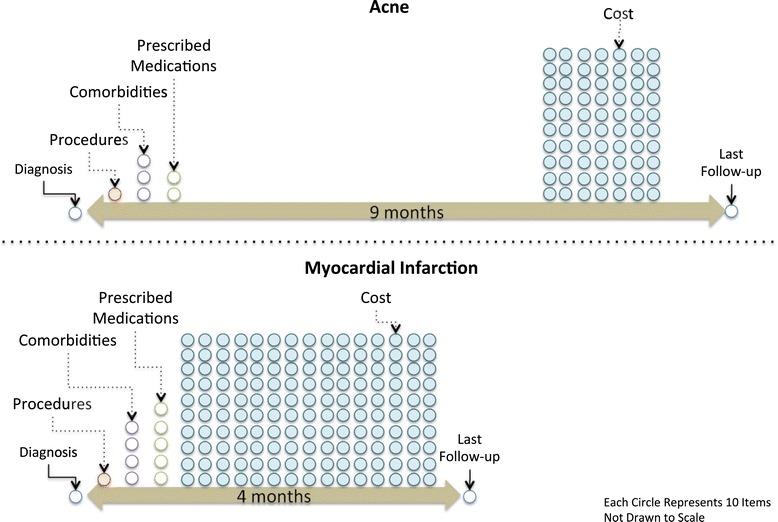
**Example showing differences between ehr manifestations of severe (Myocardial Infarction or MI) and mild (Acne) phenotypes.** Phenotype-level differences between severe and mild phenotypes are shown in Figure 1. Notice that there is very little difference between the two phenotypes if you only look at the number of procedures, comorbidities or prescribed medications. Therefore, if you use any of those three measures alone to identify severity, it would be difficult. However, if cost is used as a proxy for severity then the correct classification would be made (myocardial infarction is more severe than acne and also costs more). But if you use the treatment length then an incorrect classification of the phenotype-level severity will result (acne takes longer to treat as a result of chronicity, and therefore longer treatment length is not equal to increased phenotype-level severity). This underscores the importance of using multiple measures together as a proxy for severity, which is the approach employed by CAESAR.

*Number of comorbidities* is another useful measure for assessing phenotype severity. A similar measure is found in the Severity of Illness Index that measures the number of other conditions or problems that a given patient has at the time of their principal diagnosis. Our EHR-specific version looks at the number of distinct comorbidities per patient with a given phenotype and then averages across all of the individuals in the database with that phenotype. This average tells us the comorbidity burden associated with a given phenotype. An example is given in Figure 1 to illustrate how the number of comorbidities, medications, and treatment time can differ by phenotype severity. Note that ‘acne’ is an atypical mild phenotype as its treatment time is longer than ‘myocardial infarction’ while most mild phenotypes have shorter treatment times. Importantly, chronicity also affects treatment time, which can negate the effect that severity has on treatment time (Figure 1).

*Number of medications* is another useful measure for assessing severity. This measure is related to the previous measure (i.e., the number of comorbidities). However, it differs because some phenotypes have a large number of medications, but also a small number of comorbidities, e.g., burn injuries. Therefore, in many cases these measures will be similar but in other important instances they will differ.

*Number of procedures* is also based on a measure from the Severity of Illness Index. Because we are focused on phenotype-level severity, we computed an average number of procedures associated with each phenotype. First, we extracted the number of procedures performed per phenotype and per patient. Then we computed the average across all patients in our database yielding the average number of procedures per phenotype.

*Cost to treat phenotype* is a commonly used metric for assessing severity [25]. The Centers for Medicare and Medicaid Services released the billable rate for each procedure code per minute [26]. They also released the number of minutes each procedure typically requires. Combining these data allows us to calculate the billable amount for a given procedure [26]. The billable rates are from 2004 and they are for each Healthcare Common Procedure Coding System (HCPCS) code [26].

Since these data are only available for procedure codes (HCPCS codes are procedure codes) we calculated the total cost per patient using the procedures they were given. We determined the cost per phenotype by taking the average cost across all patients with that phenotype.

### Measures of phenotype severity and E-PSI (Ehr-phenotype severity index)

We first calculated the proportion of each measure. The sum of the proportions (there are five proportions – one for each measure) was divided by the total number of proportions (i.e., five). This final value is E-PSI, an index term based on all 5 measures given in Equation 1 where *x* is a phenotype. Therefore, E-PSI is a proportional index that incorporates treatment time, cost, number of medications, procedures, and comorbidities.

Equation 1:

***E-PSI (Phenotype x)***

$$ \begin{array}{l}=\frac{x_{cost}}{ \max (cost)} + \frac{x_{treatment\  length}}{ \max \left( treatment\  length\right)} + \frac{x_{comorbidities}}{ \max (comorbidities)}\\ {}\kern3.36em  + \frac{x_{medications}}{ \max (medications)} + \frac{x_{procedures}}{ \max (procedures)}\end{array} $$

For example the treatment time of ‘Hemoglobin SS disease with crisis’ is 1406 days. We divide this by the max treatment length of any phenotype, which is also 1406 days. This gives us the proportional treatment length of the disease or 1.00. Likewise, proportions are calculated for each of the five measures. The sum of the proportions is divided by the total number of proportions, or 5. This is E-PSI, the proportional index, for the phenotype.

We used Independent Components Analysis (ICA) [27] to visualize the relationship between E-PSI and each phenotype severity measure. Computations were performed in R (v.3.1.1).

### Reference standard development and evaluation

*Development of the Reference Standard* involved using the CUMC Clinical Data Warehouse that was transformed to the Clinical Data Model (CDM) outlined by the OMOP consortium [2]. All low prevalence phenotypes were removed, leaving behind a set of 4,683 phenotypes (prevalence of at least 0.0001). Because we are studying phenotypes manifested during the clinical encounter, we treat each distinct SNOMED-CT code as a unique phenotype. This was done because each SNOMED-CT code indicates a unique aspect of the patient state [28].

To compare results between “mild” and “severe” phenotypes, we required a reference-standard set of SNOMED-CT codes that were labeled as “mild” and “severe”. In addition, the set must be un-biased towards a particular clinical subfield (e.g., oncology or nephrology). Therefore, we developed a reference-standard set of 516 phenotypes (out of the 4,683 phenotype super-set) using a set of heuristics. All malignant cancers and accidents were labeled as “severe”; all ulcers were labeled as “mild”; all carcinomas in situ were labeled as “mild”; and most labor and delivery-related phenotypes were labeled as “mild”. Since the reference standard was created manually, the final judgment was left to the ontology expert regarding labeling a given phenotype as “mild” or “severe”. However, the ontology expert consulted with medical experts to reduce ambiguity.

*Evaluation of the Reference Standard* required soliciting volunteers to manually evaluate a subset of the reference standard (N = 7). Half of the evaluators held a Medical Degree (MD) (N = 3) and completed residency while the other half were graduate students with informatics training (N = 3) and one post-doctoral scientist. We asked each evaluator to assign phenotypes as either mild or severe. We provided each evaluator with instructions for distinguishing between mild and severe phenotypes. For example, *“*severe conditions are conditions that are life-threatening (e.g., stroke is immediately life-threatening) or permanently disabling (congenital conditions are generally considered severe unless they are easily corrected). Mild conditions may still require treatment (e.g., benign neoplasms and cysts are generally considered mild and not severe as they may not require surgery).” To ascertain the confidence that each evaluator had in making their severity assessments, we asked evaluators to denote their confidence in each severity assignment using a modified Likert scale [29] with the following 3 choices: ‘very confident’, ‘somewhat confident’ and ‘not confident’. All evaluators were provided with two coded examples and 100 randomly extracted phenotypes (from the reference standard). This evaluation set of 100 phenotypes contained 50 mild and 50 severe (labels from the reference-standard). Pair-wise agreement between each evaluator and the reference-standard was calculated using Cohen’s kappa [30,31]. Inter-rater agreement among all evaluators and the reference standard was calculated using Fleiss’s kappa [32,33].

*Evaluation of Measures at Capturing Severity* involved comparing results from “mild” and “severe” phenotypes for each severity measure. Severity measures were not normally distributed so non-parametric measures (i.e., quartiles) were used for comparisons.

### Learning phenotype-level severity classes

#### Development of the random forest classifier

CAESAR involved the unsupervised learning of classes by calculating a proximity matrix [34]. The scaled 1-proximity for each data point (in this case a phenotype) was plotted [34]. The reference standard result was then overlaid on top to determine if there was any significant clustering based on a phenotype’s class (in this case severe or mild). Clusters of severe and mild phenotypes can be used to set demarcation points for labeling a phenotype.

Using the proximity matrix also allows for discrimination among levels of severity, in addition to the binary classification of severe vs. mild. We used the randomForest package (v.4.6-10) in R (v.3.1.1) for calculations [35] and we used 1000 trees in our model. The random forest classifier, or CAESAR, takes all 5 severity measures and E-PSI (the proportional index term) as input for the model.

#### Evaluation of the random forest classifier

CAESAR was evaluated using the 516-phenotype reference standard. Sensitivity and specificity were used to assess CAESAR’s performance. The class errors for severe and mild were measured using the randomForest package [35] and compared against the out-of-bag (OOB) error rate. The randomForest algorithm uses the Gini index to measure node impurity for classification trees. The Gini impurity measure sums the probability of an item being chosen times the probability of misclassifying that item. We can assess the importance of each variable (i.e., the 5 measures and E-PSI) included in CAESAR by looking at the mean decrease in Gini. Variables with larger decreases in Gini are more important to include in CAESAR for accurate prediction.

## Results

### Assessment of phenotype severity

Severe phenotypes in general are more prevalent in EHRs because in-patient records contain “sicker” individuals when compared to the general population, which can introduce something called the Berkson bias [36]. However, in the general population mild phenotypes are often more prevalent than severe phenotypes.

**Figure 2:**
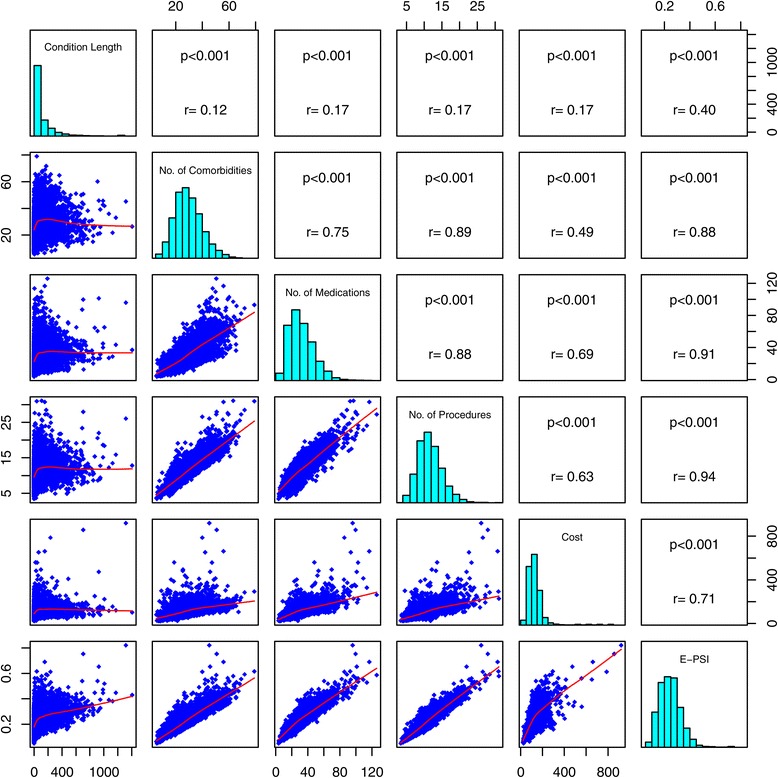
**Severity measure correlation matrix.** Histograms of each severity measure shown (along the diagonal) with pairwise correlation graphs (lower triangle) and correlation coefficients and p-values (upper triangle). Notice the condition length is the least correlated with the other measures while number of medications and number of procedures are highly correlated (r = 0.88, p < 0.001).

For condition/phenotype information we used data from CUMC EHRs, which was initially recorded using ICD-9 codes. These ICD-9 codes were mapped to SNOMED-CT codes using the OMOP CDM v.4 [2]. For this paper, we used all phenotypes (each phenotype being a unique SNOMED-CT code) with prevalence of at least 0.0001 in our hospital database. This constituted 4,683 phenotypes. We then analyzed the distribution of each of the five measures and E-PSI among the 4,683 phenotypes. Figure 2 shows the correlation matrix among the 5 severity measures and E-PSI.

Strong correlations exist between both the number of procedures and the number of medications (r = 0.88), and the number of comorbidities (r = 0.89). This indicates that there is a high degree of inter-relatedness between the number of procedures and the other severity measures. Cost was calculated using HCPCS codes alone, whereas the number of procedures measure includes both HCPCS and the ICD-9 procedure codes as defined in the OMOP CDM. Because cost was calculated using only HCPCS codes, the correlation between cost and the number of procedures was only 0.63. Also phenotype measures were increased for more severe phenotypes. This could be useful for distinguishing among subtypes of a given phenotype based on severity.

### E-PSI versus other severity measures

**Figure 3:**
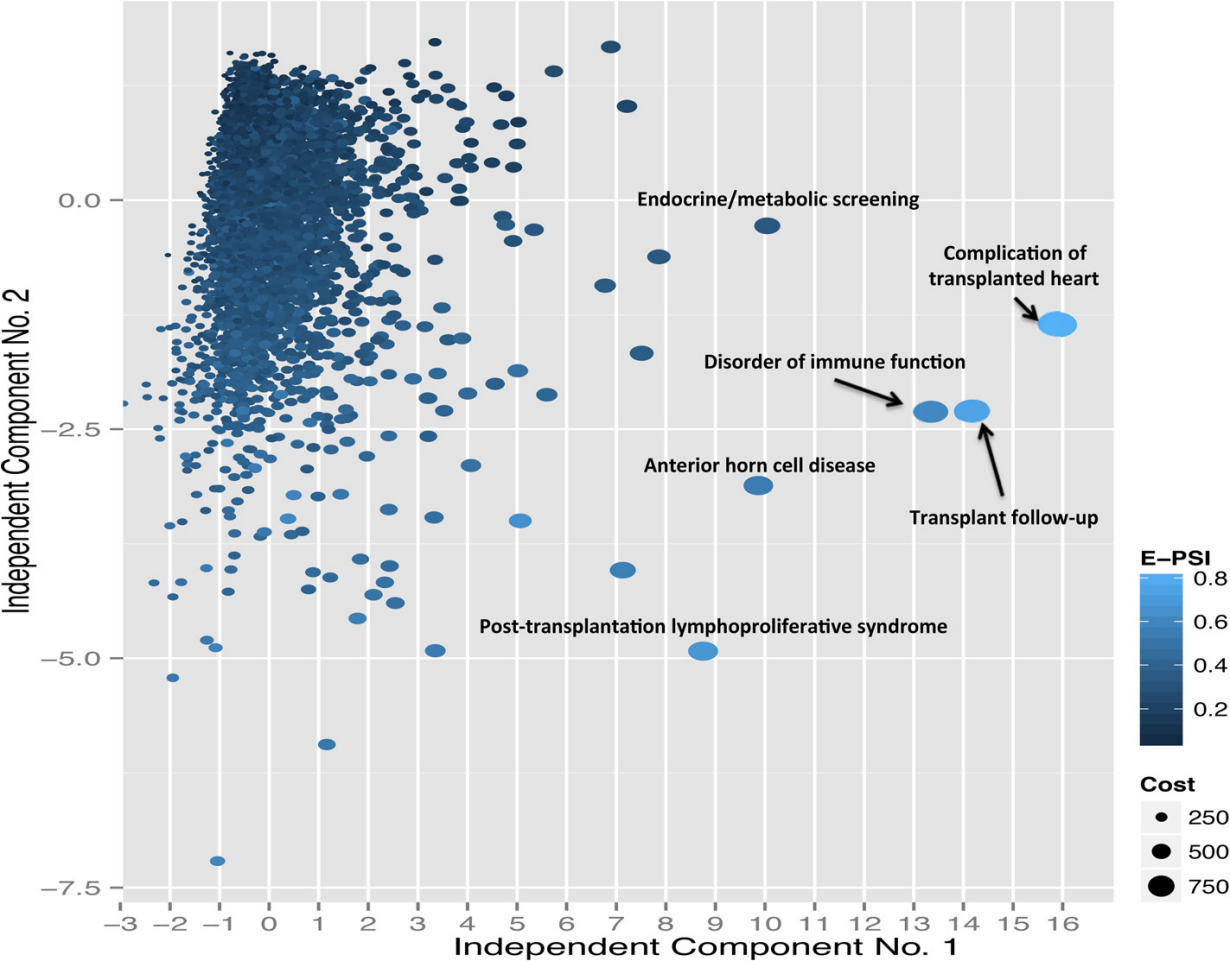
**Independent component analysis of phenotypes illustrates relationship between E-PSI and cost.** Independent Component Analysis was performed using all five severity measures and E-PSI. Phenotypes are colored by increasing E-PSI score (higher score denoted by light blue, lower score denoted by dark navy). The size indicates cost (large size indicates high cost). Phenotypes with higher E-PSI seem to be more severe; for example, ‘complication of transplanted heart’, a severe phenotype, had a high E-PSI score (and high cost). However, phenotype cost is not directly related to the E-PSI score.

We performed ICA on a data frame containing each of the five severity measures and E-PSI. The result is shown in Figure 3 with phenotypes colored by increasing E-PSI score and size denoting cost. Notice that phenotype cost is not directly related to the E-PSI score. Also phenotypes with higher E-PSI seem to be more severe (Figure 3). For example, ‘complication of transplanted heart’, a severe phenotype, had a high E-PSI score (and high cost).

Phenotypes can be ranked differently depending on the severity measure used. To illustrate this, we ranked the phenotypes using E-PSI, cost, and treatment length and extracted the top 10 given in Table 1. When ranked by E-PSI and cost, transplant complication phenotypes appeared (4/10 phenotypes), which are generally considered to be highly severe. However, the top 10 phenotypes when ranked by treatment time were also highly severe phenotypes, e.g., Human Immunodeficiency Virus and sickle cell. An ideal approach, used in CAESAR, combines multiple severity measures into one classifier.

**Table 1:** **Top 10 phenotypes ranked by severity measure**

**E-PSI**	**Cost**	**Treatment length**
*Complication of transplanted heart*	*Complication of transplanted heart*	Hemoglobin SS disease with crisis
Transplant follow-up	Transplant follow-up	*Complication of transplanted heart*
Posttransplantation lymphoproliferative syndrome	Disorder of immune function	Hemoglobin SS disease without crisis
Complication of transplanted lung	Post-transplantation lymphoproliferative syndrome	Exstrophy of bladder sequence
Complication of hemodialysis	Anterior horn cell disease	Factor IX deficiency
Disorder of immune function	Endocrine/metabolic screening	Complication of transplanted kidney
Complication of renal dialysis	Myocardial degeneration	Type II diabetes mellitus - poor control
Disorder of transplanted bone marrow	APL - Acute promyelocytic leukaemia	Sickle cell-hemoglobin C disease without crisis
Arrested development following proteincalorie malnutrition	Isolated (Fiedler’s) myocarditis	HIV - Human immunodeficiency virus infection
Serratia septicaemia	Complication of transplanted lung	Osteoarthritis

‘Complication of transplanted heart’ appears in the top 10 phenotypes when ranked by all three-severity measures (*italicized* in Table 1). This is particularly interesting because this phenotype is both a complication phenotype and transplant phenotype. By being a complication the phenotype is therefore a severe subtype of another phenotype, in this case a heart transplant (which is actually a procedure). Heart transplants are only performed on sick patients; therefore this phenotype is always a subtype of another phenotype (e.g., coronary arteriosclerosis). Hence ‘complication of transplanted heart’ is a severe subtype of multiple phenotypes (e.g., heart transplant, and the precursor phenotype that necessitated the heart transplant – coronary arteriosclerosis).

### Evaluation of severity measures

*Development of the Reference Standard* severe and mild SNOMED-CT codes involved using a set of heuristics with medical guidance. Phenotypes were considered severe if they were life threatening (e.g., ‘stroke’) or permanently disabling (e.g., ‘spina bifida’). In general, congenital phenotypes were considered severe unless easily correctable. Phenotypes were considered mild if they generaly require routine or non-surgical (e.g., ‘throat soreness’) treatment.

Several heuristics were used: 1) all benign neoplasms were labeled as mild; 2) all malignant neoplasms were labeled as severe; 3) all ulcers were labeled as mild; 4) common symptoms and conditions that are generally of a mild nature (e.g., ‘single live birth’, ‘throat soreness’, ‘vomiting’) were labeled as mild; 5) phenotypes that were known to be severe (e.g., ‘myocardial infarction’, ‘stroke’, ‘cerebral palsy’) were labeled as severe. The ultimate determination was left to the ontology expert for determining the final classification of severe and mild phenotypes. The ontology expert consulted with medical experts when deemed appropriate. The final reference standard consisted of 516 SNOMED-CT phenotypes (of the 4,683 phenotypes). In the reference standard, 372 phenotypes were labeled as mild and 144 were labeled as severe.

*Evaluation of the Reference Standard* was performed using volunteers from the Department of Biomedical Informatics at CUMC. Seven volunteers evaluated the reference standard including three MDs with residency training, three graduate students with informatics experience and one post-doc (non-MD). Compensation was commensurate with experience (post-docs received $15 and graduate students received $10 Starbucks gift cards).

We excluded two evaluations from our analyses: one because the evaluator had great difficulty with the medical terminology, and the second because the evaluator failed to use the drop-down menu provided as part of the evaluation. We calculated the Fleiss kappa for inter-rater agreement among the remaining 5 evaluations and found evaluator agreement was high (k = 0.716). The individual results for agreement between each evaluator and the reference standard were kappa equal to 0.66, 0.68, 0.70, 0.74, and 0.80. Overall, evaluator agreement (k = 0.716) was sufficient for comparing two groups (i.e., mild and severe) and 100% agreement was observed between all five raters and the reference-standard for 77 phenotypes (of 100).

**Figure 4:**
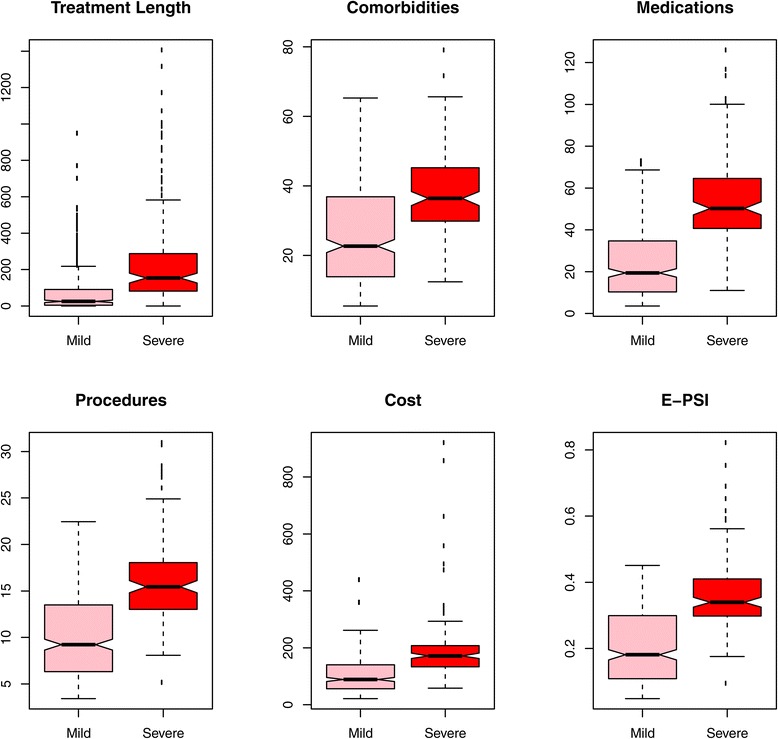
**Differences in severity measures and e-psi for mild vs. severe phenotypes.** The distribution of each of the 6 measures used in CAESAR is shown for severe and mild phenotypes. Severity assignments were from our reference standard. Using the Wilcoxon Rank Sum Test, we found statistically significant differences between severe and mild phenotypes across all 6 measures (p < 0.001). Severe phenotypes (dark red) having higher values for each of the six measures than mild phenotypes. The least dramatic differences were observed for cost and number of comorbidities while the most dramatic difference was for the number of medications.

*Evaluation of Measures at Capturing Severity* was performed by comparing the distributions of all 6 measures between severe and mild phenotypes in our 516-phenotype reference standard. Results are shown in Figure 4. Increases were observed for severe phenotypes across all measures. We performed the Wilcoxon Rank Sum Test to assess significance of the differences between severe vs. mild phenotypes shown in Figure 4. The p-values for each comparison were <0.001.

### Unsupervised learning of severity classes

#### Development of the random forest classifier

CAESAR used an unsupervised random forest algorithm (randomForest package in R) that required E-PSI and all 5-severity measures as input. We ran CAESAR on all 4,683 phenotypes and then used the 516-phenotype reference standard to measure the accuracy of the classifier.

#### Evaluation of the random forest classifier

**Figure 5:**
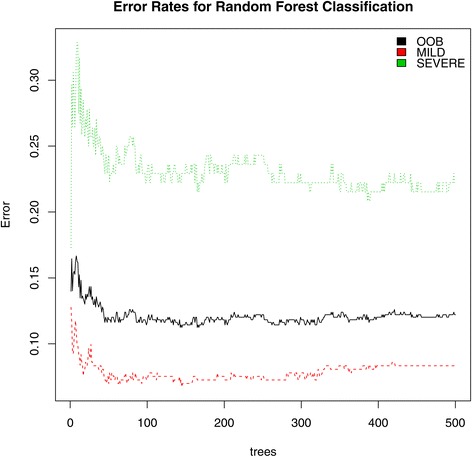
**CAESAR error rates.** Error rates for CAESAR’s random forest classified are depicted with severe denoted by the green line, mild denoted by the red line and out-of-bag (OOB) error denoted by the black line. CAESAR achieved a sensitivity = 91.67 and specificity = 77.78 indicating that it was able to discriminate between severe and mild phenotypes. CAESAR was able to detect mild phenotypes better than severe phenotypes.

CAESAR achieved a sensitivity = 91.67 and specificity = 77.78 indicating that it was able to discriminate between severe and mild phenotypes. CAESAR was able to detect mild phenotypes better than severe phenotypes as shown in Figure 5.

The Mean Decrease in Gini (MDG) measured the importance of each severity measure in CAESAR. The most important measure was the number of medications (MDG = 54.83) followed by E-PSI (MDG = 40.40) and the number of comorbidities (MDG = 30.92). Cost was the least important measure (MDG = 24.35).

**Figure 6:**
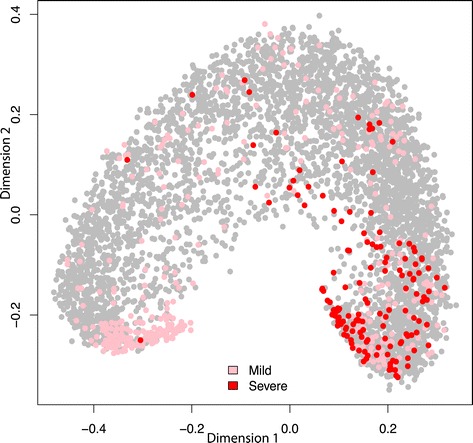
**Classification result from CAESAR showing all 4,683 phenotypes (gray) with severe (red) and mild (pink) phenotype labels from the reference standard.** All 4,683 phenotypes plotted using CAESAR’s dimensions 1 and 2 of the scaled 1-proximity matrix. Severe phenotypes are colored red, mild phenotypes are colored pink and phenotypes not in the reference standard are colored gray. Notice that most of the severe phenotypes are in the lower right hand portion of the plot while the “mild” space is found in the lower left hand portion.

CAESAR used all 4,683 phenotypes plotted on the scaled 1-proximity for each phenotype [34] shown in Figure 6 with the reference standard overlaid on top. Notice that phenotypes cluster by severity class (i.e., mild or severe) with a “mild” space (lower left) and a “severe” space (lower right), and phenotypes of intermediate severity in between.

However, three phenotypes are in the “mild” space (lower left) of the random forest model (Figure 6). These phenotypes are ‘allergy to peanuts’, ‘suicide-cut/stab’, and ‘motor vehicle traffic accident involving collision between motor vehicle and animal-drawn vehicle, driver of motor vehicle injured’. These phenotypes are probably misclassified because they are ambiguous (in the case of the motor vehicle accident, and the suicide cut/stab) or because the severity information may be contained in unstructured EHR data elements (as could be the case with allergies).

Using the proximity matrix also allows further discrimination among severity levels beyond the binary mild vs. severe classification. Phenotypes with ambiguous severity classifications appear in the middle of Figure 6. To identify highly severe phenotypes, we can focus only on phenotypes contained in the lower right hand portion of Figure 6. This reduces the phenotype selection space from 4,683 to 1,395 phenotypes (~70% reduction).

We are providing several CAESAR files for free download online at http://caesar.tatonettilab.org. These include, the 516-phenotype reference-standard used to evaluate CAESAR, the 100-phenotype evaluation set given to the independent evaluators along with the instructions, and the 4,683 conditions with their E-PSI scores and the first and second dimensions of the 1-proximity matrix (shown in Figure 6). This last file also contains two subset tables containing the automatically classified “mild” and “severe” phenotypes and their scores.

## Discussion

Using the patient-specific severity index as a backbone [19], we identified five measures of EHR-specific phenotype severity that we used as input for CAESAR. Phenotype-level severity differs from patient-level severity because it is an attribute of the phenotype itself and can be used to rank phenotypes. Using CAESAR, we were able to reduce our 4,683-phenotype set (starting point) to 1,395 phenotypes with high severity and prevalence (at least 0.0001) reducing the phenotype selection space by ~70%. Severe phenotypes are highly important for CER because they generally correlate with lower survival outcomes, lost-productivity, and have an increased cost burden. In fact, patients with severe heart failure tend to have bad outcomes regardless of the treatment they receive [37]. Therefore understanding the severity of each condition is important before performing CER and having a complete list of severe phenotypes would be greatly beneficial.

Additionally, developing a classification algorithm that is biased towards identifying more severe over mild phenotypes is optimal, as it would enable detection of phenotypes that are crucial for public health purposes. Active learning methods that favor detection of severe phenotypes were proven successful in a subsequent study [38].

CAESAR uses an integrated severity measure approach, which is better than using any of the other measures alone, e.g., cost, as each severity measure has its own specific bias. It is well known that cosmetic procedures, which by definition treat mild phenotypes, are high in cost. If cost is used as a proxy for severity it could introduce many biases towards phenotypes that require cosmetic procedures (e.g., crooked nose) that are of little importance to public health. Also some cancers are high in cost but low in mortality (and therefore severity), a good example being non-melanoma skin cancer [39]. Therefore, by including multiple severity measures in CAESAR we have developed a method that is robust to these types of biases.

Another interesting finding was that cancer-screening codes tend to be classified as severe phenotypes by CAESAR even though they were generally considered as mild in the reference standard. The probable cause for this is that screening codes, e.g., ‘screening for malignant neoplasm of respiratory tract’, are generally only assigned by physicians when cancer is one of the differential diagnoses. In this particular situation the screening code, while not an indicator of the disease itself, is indicative of the patient being in an abnormal state with some symptoms of neoplastic presence. Although not diagnoses, screening codes are indicative of a particular manifestation of the patient state, and therefore can be considered as phenotypes. This finding is also an artifact of the EHR, which records the patient state [8], which does not always correlate with the “true” phenotype [5,28].

Importantly, CAESAR may be useful for distinguishing among subtypes of a given phenotype if one of the characteristics of a subtype involves severity. For example, the severity of Gaucher disease subtypes are difficult to capture at the patient-level [40]. This rare phenotype would benefit greatly from study using EHRs where more patient data exists. Using CAESAR may help in capturing the phenotype-level severity aspect of this rare phenotype, which would help propel the utility of using EHRs to study rare phenotypes [41] by providing accurate severity-based subtyping.

CAESAR is directly relevant to the efforts of the Observational Health Data Sciences and Informatics consortium (OHDSI), which is a continuation of OMOP. OHDSI is an international network focused on observational studies using EHRs and other health record systems. Their original motivation was to study post-market effects of pharmaceutical drugs [42] based on their pharmaceutical partnerships. To this end, a severity-based list of ranked phenotypes would be beneficial for assessing the relative importance of various post-marketing effects (e.g., nausea is mild, arrhythmia is severe).

Other phenotyping efforts would also benefit from CAESAR including the eMERGE network [3], which seeks to carefully define phenotypes of interest for use in PheWAS studies. So far they have classified 20 phenotypes. Having a ranked list of phenotypes would help eMERGE to rank prospective phenotypes, thereby allowing them to select more severe phenotypes for further algorithm development efforts.

There are several limitations to this work. The first is that we used CUMC data when calculating four of the severity measures. Because we used only one institution’s data, we have an institution-specific bias. However, since CAESAR was designed using the OMOP CDM, it is portable for use at other institutions that conform to the OMOP CDM. The second limitation is that we did not use clinical notes to assess severity. Some phenotypes, e.g., ‘allergy to peanuts’, may be mentioned more often in notes than in structured data elements. For such phenotypes, CAESAR would under estimate their severity. The third limitation is that we only used procedure codes to determine phenotype cost. Therefore, phenotypes that do not require procedures will appear as low cost phenotypes even though they may have other costs, e.g., medications.

Future work involves investigating the inter-relatedness of our severity measures and determining the temporal factors that affect these dependencies. We also plan to investigate the inter-dependency of phenotypes (e.g., ‘blurred vision’ is a symptom of ‘stroke’, but both are treated as separate phenotypes) and determine the utility of our severity measures for distinguishing between phenotypes and their subtypes.

Another potentially interesting extension of our work could involve utilizing the semantics of SNOMED, specifically their phenotype/subtype relations, to explore CAESAR’s severity results. Because we chose SNOMED to represent each phenotype, we can leverage SNOMED’s semantics to further probe the relationship between severity and disease. Perhaps some of the phenotypes with ambiguous severity (middle of Figure 6) occurred because their disease subtypes can be either mild or severe (we can assess this using SNOMED’s hierarchical structure). However, leveraging the semantics of concepts for severity classification is a complex area [43], which will likely require additional methods to tackle. Hopefully these topics can be explored in future by ourselves or others.

## Conclusions

This paper presents CAESAR, a method for classifying severity from EHRs. CAESAR takes several known measures of severity: cost, treatment time, number of comorbidities, medications, and procedures per phenotype, and a proportional index term as input into a random forest algorithm that classifies each phenotype as either mild or severe. Using a reference standard that was validated by medical experts (k = 0.716), we found that CAESAR achieved a sensitivity of 91.67 and specificity of 77.78 for severity detection. CAESAR reduced our 4,683-phenotype set (starting point) to 1,395 phenotypes with high severity. By characterizing phenotype-level severity using CAESAR, we can identify phenotypes worthy of study from EHRs that are of particular importance for CER and public health.

## Abbreviations

CER: Comparative Effectiveness Research
HOI: Health Outcomes of Interest
OMOP: Observational Medical Outcomes Partnership
eMERGE: The Electronic Medical Records and Genomics Network
PheWAS: Phenome-Wide Association
EHRs: Electronic Health Records
HPO: Human Phenotype Ontology
SNOMED-CT: Systemized Nomenclature of Medicine – Clinical Terms
CAESAR: Classification Approach for Extracting Severity Automatically from Electronic Health Records
CUMC: Columbia University Medical Center
HCPCS: Healthcare Common Procedure Coding System
E-PSI: Ehr-phenotype severity index
ICA: Independent Components Analysis
CDM: Clinical Data Model
MD: Medical Degree
OOB: Out-of-bag error rate
MDG: Mean Decrease in Gini
OHDSI: Observational Health Data Sciences and Informatics consortium
ICD-9: International classifcation of diseases, 9th revision
